# Functional characterization of maternally accumulated hydrolases in the mature oocytes of the vector *Rhodnius prolixus* reveals a new protein phosphatase essential for the activation of the yolk mobilization and embryo development

**DOI:** 10.3389/fphys.2023.1142433

**Published:** 2023-02-27

**Authors:** Elisa de Almeida, Uilla Dittz, Jéssica Pereira, Ana B. Walter-Nuno, Gabriela O. Paiva-Silva, Marco A. Lacerda-Abreu, Jose R. Meyer-Fernandes, Isabela Ramos

**Affiliations:** ^1^ Instituto de Bioquímica Médica Leopoldo de Meis. Universidade Federal do Rio de Janeiro, Rio de Janeiro, Brazil; ^2^ Instituto Nacional de Ciência e Tecnologia em Entomologia Molecular—INCT-EM/CNPq, Rio de Janeiro, Brazil

**Keywords:** yolk degradation, vector biology, oogenesis, *Rhodnius prolixus* (Insecta), RNAi

## Abstract

Yolk biogenesis and consumption have been well conserved in oviparous animals throughout evolution. Most egg-laying animals store yolk proteins within the oocytes’ yolk granules (Ygs). Following fertilization, the Ygs participate in controlled pathways of yolk breakdown to support the developing embryo’s anabolic metabolism. While the unfolding of the yolk degradation program is a crucial process for successful development in many species, the molecular mechanisms responsible for yolk mobilization are still mysterious and have mostly not been explored. Here, we investigate the functional role of the oocyte maternally accumulated mRNAs of a protein phosphatase (PP501) and two aspartic proteases (cathepsin-D 405, CD405 and cathepsin-D 352, CD352) in the yolk degradation and reproduction of the insect vector of Chagas disease *Rhodnius prolixus*. We found that PP501 and CD352 are highly expressed in the vitellogenic ovary when compared to the other organs of the adult insect. Parental RNAi silencing of PP501 resulted in a drastic reduction in oviposition and increased embryo lethality whereas the silencing of CD352 resulted only in a slight decrease in oviposition and embryo viability. To further investigate the PP501-caused high reproduction impairment, we investigated the Ygs biogenesis during oocyte maturation and the activation of the yolk degradation program at early development. We found that the Ygs biogenesis was deficient during oogenesis, as seen by flow cytometry, and that, although the PP501-silenced unviable eggs were fertilized, the Ygs acidification and acid phosphatase activity were affected, culminating in a full impairment of the yolk proteins degradation at early embryogenesis. Altogether we found that PP501 is required for the oocyte maturation and the activation of the yolk degradation, being, therefore, essential for this vector reproduction.

## Introduction

In oviparous animals, the processes of yolk biogenesis and consumption have been well-conserved through evolution. Most egg-laying animals store yolk proteins within the oocytes, which provide nutrients and energy for embryo development. A highly specialized oocyte cytoplasm made up of maternal mRNAs, ribosomes, mitochondria, and primarily a group of organelles known as yolk granules (Ygs) is produced as a result of yolk accumulation. Following fertilization, the Ygs participate in controlled pathways of yolk breakdown to support the developing embryo’s anabolic metabolism. Buildup and degradation of the yolk are thus crucial processes for successful development in many species. However, the molecular systems and mechanisms involved in the controlled mobilization of the yolk throughout development are still mysterious and have mostly not been studied (Gilbert and Barresi, 2018; Ramos et al., 2022).

A group of enzymes that will be in charge of mobilizing the yolk throughout development is also stored in the Ygs along with the yolk proteins. The most important discovery about the Ygs function in terms of molecular mechanism is that some of them resemble modified lysosomes which can adjust their pH to enable the activation of acid hydrolases. Pasteels, 1966, proposed the lysosomal nature of the Ygs and their latency more than 50 years ago (Pasteels, 1966). Since then, several species have validated his findings, although the precise rules governing the organelle’s programmed acidification and programmed yolk degradation are still unknown.

The primary yolk protease in ticks is a cysteine protease that resembles a cathepsin L and is kept as a latent proenzyme that is inactive at moderately acidic pHs (Fagotto, 1990). As primary degrading enzymes, acidic cysteine proteinases are also found in the yolk of sea urchins (Mallya et al., 1992), the german cockroach *Blattella germanica* (Nordin et al., 1991), the silk moth *Bombyx mori* (Kageyama et al., 1981; Takahashi et al., 1992; Takahashi et al., 1996) and the fruit fly *Drosophila melanogaster* (Medina and Vallejo, 1989). A carboxypeptidase (Cho et al., 1991) and a cathepsin B-like protease (Cho et al., 1999) have both been identified in the yellow fever mosquito *Aedes aegypti*. Acid proteases were not the only enzymes discovered in various models; yolk-targeting acid phosphatases, glycosidases, and ribonucleases were also mentioned (Purcell et al., 1988; Nussenzveig et al., 1992; Liao and Wang, 1994; Takahashi et al., 1996; Ribolla et al., 2001; Fialho et al., 2002; Fialho et al., 2005; Oliveira and Machado, 2006). Although it is thought that these hydrolytic enzymes are predominantly of maternal origin and are received either from the nurse and/or follicular cells or by endocytosis, the exact source of these enzymes is unknown. In addition, it is believed that other regulatory mechanisms must exist to allow yolk proteolysis, as regulating the pH in the lumen of the Ygs by itself is probably insufficient to permit a fine-tuned yolk breakdown (Fagotto, 1995; Ramos et al., 2022).

A number of fundamental aspects of insect physiology were discovered in the strictly hematophagous hemipteran *R. prolixus* early in the 20th century (Wigglesworth, 1934a; Wigglesworth, 1934b; Wigglesworth, 1951; Edwards, 1998; Davey, 2007; Wigglesworth, 2012). Also, this insect is significant for human health as the etiological vector of the neglected tropical disease Chagas Disease (Chagas, 1909), which is endemic in Central and South America (World Health Organization, 2019). More than 8 million people are already affected by Chagas disease, and estimates indicate that climate change and globalization will cause its endemic region to grow considerably more (Eberhard et al., 2020). In *Rhodnius prolixus*, a pepstatin-sensitive aspartic protease (cathepsin-D) activity has been identified as essential for *in vitro* yolk protein degradation (Nussenzveig et al., 1992). In addition, an acid phosphatase involved in yolk protein dephosphorylation was isolated from the egg homogenates, and its activity has been characterized during embryo development (Fialho et al., 2002). Interestingly, it has been observed that activation of the acid phosphatase activity must occur before activation of the cathepsin-D (Gomes et al., 2010), and that inorganic polyphosphate acts as an inhibitor of the aspartic protease activity (Fialho et al., 2005), indicating that the interaction between these enzymes and phosphorylated molecules is crucial for controlling yolk mobilization.

In this work, we explored the *R. prolixus* ovary transcriptome (Coelho et al., 2021) and analyzed one protein phosphatase (PP501) and two aspartyl proteases (cathepsin-D) (CD352 and CD405) mRNAs maternally accumulated in chorionated oocytes. Using Reverse Transcription Quantitative PCR (RT-qPCR), PP501 and CD352 were found to be highly expressed in the ovaries when compared to their expression in the midgut, fat body, and flight muscle of adult vitellogenic females, whereas CD405 was highly expressed in the midgut when compared to the other organs. Parental RNAi silencing of PP501 resulted in highly compromised oviposition rates and embryo lethality, whereas the silencing of CD352 generated only minor decreases in oviposition and embryo viability. Interestingly, we found that PP501-silenced eggs, although fertilized, presented impaired Ygs acidification and inhibited yolk-targeting acid phosphatase activity, culminating in failed activation of the yolk proteins degradation. Altogether, we found that PP501 is a key enzyme for the signals that coordinate yolk degradation being crucial for this vector reproduction.

## Materials and methods

### Bioinformatics

The sequences of *R. prolixus* PP501 (RPRC002352), CD352 (RPRC006759), and CD405 (RPRC006028) were obtained from the *R. prolixus* genome and transcriptome databases (Rpro C3.2) from Vector Base (www.vectorbase.org) (Ribeiro et al., 2014; Giraldo-Calderón et al., 2015; Mesquita et al., 2015). Conserved domains were predicted using PFAM (http://pfam.xfam.org/) (Mistry et al., 2021).

### Insects

Insects were maintained at a 28°C ± 2°C controlled temperature, relative humidity of 70%–80%, and 12/12 h light and dark cycles. All females used in this work were obtained from our insectarium where mated females are fed for the first time (as adult insects) in live-rabbit blood 14–21 days after the fifth instar nymph to adult ecdysis. After the first blood feeding, all adult insects in our insectarium are fed every 21 days. For all experiments, mated females of the second or third blood feeding were used, and dissections were carried out on different days after the blood meal depending on the experiment.

### Ethics statement

All animal care and experimental protocols were approved by guidelines of the institutional care and use committee (Committee for Evaluation of Animal Use for Research from the Federal University of Rio de Janeiro, CEUA-UFRJ #01200.001568/2013-87, order number 155/13), under the regulation of the national council of animal experimentation control (CONCEA). Technicians dedicated to the animal facility conducted all aspects related to animal care under strict guidelines to ensure careful and consistent animal handling.

### Dissection of the ovary parts, follicles, and chorionated oocytes

The different parts of the ovariole were carefully dissected in phosphate-buffered saline (PBS) 137 mM NaCl, 2.7 mM KCl, 10 mM Na_2_HPO_4_, and 1.8 mM KH_2_PO_4_, pH 7.4 using fine tweezers and dissecting scissors under the stereo microscope according to (Huebner and Injeyan, 1980), and the structures (tropharium, previtellogenic and vitellogenic follicles, and chorionated oocytes) were classified by length and morphology according to (Bjornsson and Huebner, 2004; Nunes-da-fonseca et al., 2017). When chorionated oocytes were used, dissection was performed 7–12 days after the blood meal. All other organs and ovary parts were dissected 7 days after the blood meal.

### Extraction of RNA and cDNA synthesis

All samples were homogenized in Trizol reagent (Invitrogen) for total RNA extraction. Reverse transcription reaction was carried out using the High-Capacity cDNA Reverse Transcription Kit (Applied Biosystems) using 1 µg of total RNA after RNase-free DNase I (Invitrogen) treatment, Multiscribe Reverse Transcriptase enzyme (2.5 U/µL) and random primers for 10 min at 25°C followed by 2 h of incubation at 37°C. As a control for the DNAse treatment efficiency, we performed control reactions without the enzyme followed by testing the capacity of amplification by PCR.

### PCR/RT-qPCR

Specific primers for the *R. prolixus* PP501, CD405, and CD352 sequences were designed to amplify 142 base pairs (bp), 138 bp, and 195 bp fragments, respectively, in a PCR using the following cycling parameters: 10 min at 95°C, followed by 35 cycles of 15 s at 95°C, 45 s at 52°C and 30 s at 72°C and a final extension of 15 min at 72°C. Amplifications were observed in 2% agarose gels. Quantitative PCR (qPCR) was performed in a StepOne Real-Time PCR System (Applied Biosystems) using SYBR Green PCR Master Mix (Applied Biosystems) under the following conditions: 10 min at 95°C, followed by 40 cycles of 15 s at 95°C and 45 s at 60°C. The cDNAs were diluted 10X and used in the reactions. To exclude non-specific amplification, blank reactions replacing the template (cDNA) for water were performed in all experiments. The relative expressions were calculated using the delta C_t_ (cycle threshold) obtained using the reference gene 18S (RPRC017412) and expressed as 2^−ΔΔCT^. According to the minimum information for publication of quantitative RT-qPCR experiments (MIQE) Guidelines, normalization against a single reference gene is acceptable when the investigators present clear evidence that confirms its invariant expression under the experimental conditions (Bustin et al., 2009). The use of the ribosomal gene 18S has been described before for *R. prolixus* (Majerowicz et al., 2011), and Supplementary Figure S1 shows its invariant expression in our experimental conditions. All primers are described in Supplementary Table S1.

### RNAi silencing

dsRNA was synthesized by MEGAScript RNAi Kit (Ambion Inc.) using primers for PP501 and CD352 specific gene amplification (Supplementary Table S1). Unfed adult females were injected between the second and third thoracic segments using a 10 µl Hamilton syringe with 1 µg dsRNA (diluted in 1 µl of water) and fed 2 days later. Knockdown efficiency was confirmed by qPCR at 7 days after blood meal. The bacterial *MalE* gene was used as a control dsRNA (Hansen et al., 2005). Adult females injected with dsRNA were fed and transferred to individual vials. The mortality rates and the number of eggs laid by each individual were recorded daily and weekly, respectively. Additional measurements are described below.

### Egg homogenates and sodium dodecyl-sulfate polyacrylamide gel electrophoresis (SDS-PAGE)

Control and silenced eggs were collected at day 1 (24 h after oviposition) and day 4 (96 h after oviposition) of embryogenesis. Pools of four eggs were homogenized using a plastic pestle in 100 µl of PBS. Protein concentrations of the supernatants were determined by the Lowry method (Lowry et al., 1951). The samples were directly used, and 30 µg of total protein was loaded in each lane of a 10% SDS-PAGE. Gels were stained with silver nitrate (Merril et al., 1981).

### Ygs suspension and flow cytometry

Suspensions of Ygs were obtained as previously described (Vieira et al., 2021). Briefly, recently dissected chorionated oocytes were gently disrupted in PBS (2 oocytes in 250 µl of buffer) using a plastic pestle. The population profiles of the yolk organelles were acquired on a FACS Calibur instrument (BD Bioscience, USA) powered by CellQuest Pro software v5.1 and analyzed using Flowing Software 2.5.1. The Ygs suspensions were also observed under DIC microscopy in a Zeiss Axio Imager D2.

### Egg homogenates and assays for acid phosphatase activity

For the determination of acid phosphatase-specific activity, 4-day eggs were disrupted in PBS using a plastic pestle and were subjected to three cycles of freeze and thaw. Protein concentrations of the supernatants were determined by the Lowry method (Lowry et al., 1951). For each fraction, aliquots containing 30 µg of protein were assayed at 28°C against 4 mM p-nitrophenylphosphate (*p-*NPP) in the following reaction medium: 20 mM sodium acetate, pH 4.0, 1 mM DTT, and 1 mM EDTA. Reactions were stopped after 1 h by the addition of 0.2 M NaOH (corresponding to 10% of the total reaction volume) and each sample, containing the reaction hydrolysis product (p-nitrophenol, p-NP), had its absorbance measured at 405 nm in a Thermomax microplate reader (Fialho et al., 2002; Ramos et al., 2007). The phosphatase activity was calculated by subtracting the non-specific *p-*NPP hydrolysis measured in the absence of protein. To determine the concentration of released p-NP, a p-NP curve was plotted and used as a standard (Rodrigues et al., 1999).

### Egg-fertilization assay

To determine fertilization in control and silenced eggs, a PCR was performed targeting a male-specific DNA fragment (chromosome Y) (GenBank: JX559072.1), as previously reported by (Walter-Nuno et al., 2013). Briefly, freshly laid eggs from control and silenced individuals were collected and genomic DNA was phenol extracted and precipitated with 100% ethanol and 2M ammonium acetate. Purified genomic DNA samples were used as templates for amplification using specific primers. As positive control, the fat body of adult male insects was used. Eggs laid by non-mated females were used as a negative control.

### Ygs acidification assay

Day-4 eggs (96 h after oviposition) were extracted and incubated in the dark for 10 min in PBS containing 5 μg/ml of the acid marker acridine orange (AO), as previously reported (Ramos et al., 2007). After incubation, the Ygs suspensions were deposited on glass slides and observed at an excitation wavelength of 418 nm in a Zeiss Axioimager d2 epifluorescence microscope equipped with a fluorescein filter set and a TK-1270 JVC color video camera.

### Statistics

Data was tested for normality distribution using the Shapiro-Wilk normality test. When the data passed the normality test, we used Student’s t-Test for the comparison of two different conditions and One-way ANOVA or Two-way ANOVA followed by Tukey’s multiple comparisons for the comparison among more than two conditions. When the data did not pass the normality test, the non-parametric Kruskal-Wallis test followed by Dunn’s multiple comparisons test were performed. Log-rank (Mantel-Cox) test was performed for the survival experiments. Differences were considered significant at *p* < 0.05. All statistical analyses were performed using Prism 7.0 software (GraphPad Software).

## Results

### PP501 and CD352 maternal mRNAs are accumulated in the ovaries and developing oocytes

**FIGURE 1:**
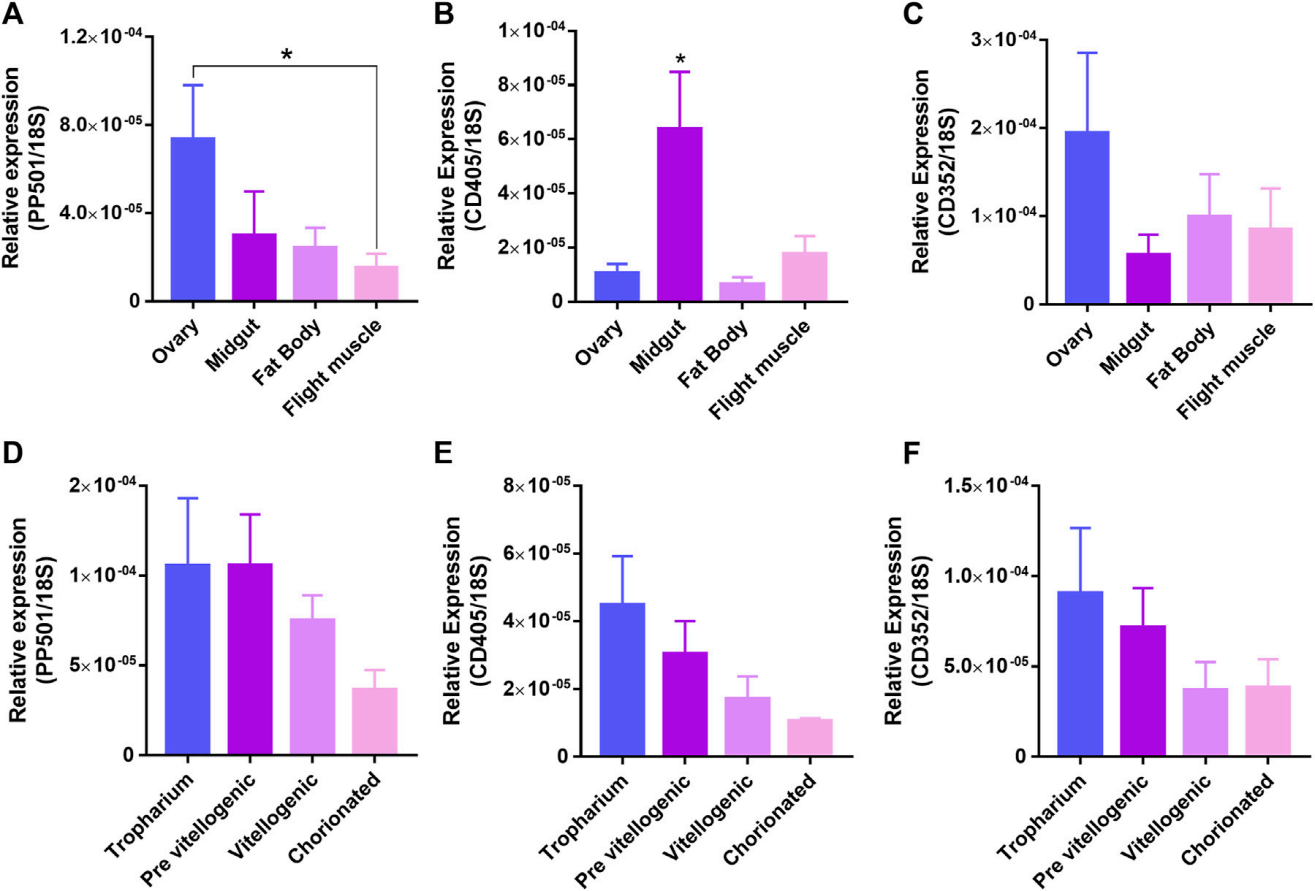
PP501 and CD352 are highly expressed in the ovaries whereas CD405 is highly expressed in the midgut of vitellogenic females. **(A–C)**. RT-qPCR showing the relative expression of PP501, CD405 and CD352 in different organs. Graphs show mean ± SEM (n = 9). *p < 0.05, Kruskal-Wallis. **(D–F)**. RT-qPCR showing the relative expression of PP501, CD405 and CD352 during oogenesis of vitellogenic females. Graphs show mean ± SEM (n = 6). *p < 0.05, Kruskal-Wallis. All organs were dissected 7 days after the blood meal. The relative expression was quantified using the ∆CT method using Rp18S as endogenous control.

The gene PP501 (RPRC002352) encodes the catalytic subunit of a predicted serine-threonine protein phosphatase (EC 3.1.3.16), presenting the characteristic domains: N-terminal serine-threonine protein phosphatase domain (*STPPase_N*, pfam: 19,891) and calcineurin-like phosphoserine phosphatase (*Metallophos*, Pfam: 00,149) (Supplementary Figure S21). The genes CD352 (RPRC006759) and CD405 (RPR006028) encode predicted aspartyl proteases, containing the major aspartic protease conserved domain (*Asp*, Pfam: PF00026) (Supplementary Figure S2). Based on their Reads Per Kilobase Million (RPKM) levels, those genes were found within the 5% most abundant mRNAs in the transcriptome of chorionated (mature) oocytes of *R. prolixus* previously published by Coelho et al., 2021 *,* and, for this reason, we decided to further explore their role during oocyte maturation and embryo development. Although high individual variations were observed, RT-qPCRs showed that the ovary of *R. prolixus* presents a tendency of higher expression of PP501 and CD352 when compared to the midgut, fat body and fligh muscle (Figures 1A, C), whereas CD405 was found to be 5x more expressed in the midgut than in the other organs (Figure 1B). Within the ovary, the highest mRNA levels of PP501, CD405, and CD352 were detected in the tropharium (the structure that harbors the germ cell cluster and nurse cells) and early previtellogenic follicles, which presented a trend of approximately 2x the mRNA levels detected in the vitellogenic follicles and chorionated oocytes (Figures 1D–F).

### Parental RNAi silencing of PP501 and CD352 generates decreased oviposition and increased embryo lethality

**FIGURE 2:**
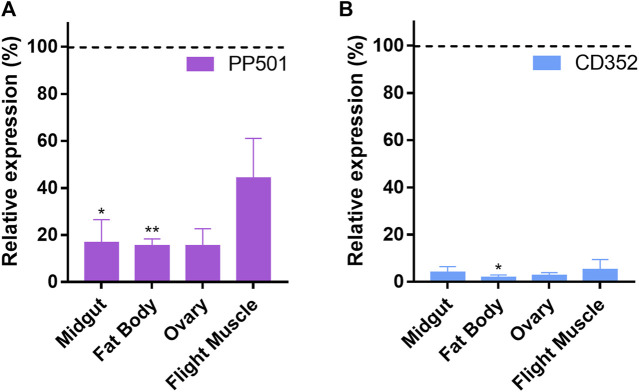
RNAi knockdown of PP501 and CD352 is efficient and systemic. **(A, B)**. RT-qPCR showing the relative expression levels of PP501 **(A)** and CD352 **(B)** in the ovary, fat body, midgut and flight muscle of silenced females when compared to control (dsMal) levels (represented by the dashed lines). The organs were dissected 7 days after the blood meal (n = 3). *p < 0.05, **p < 0.01, *t*-test.

**FIGURE 3:**
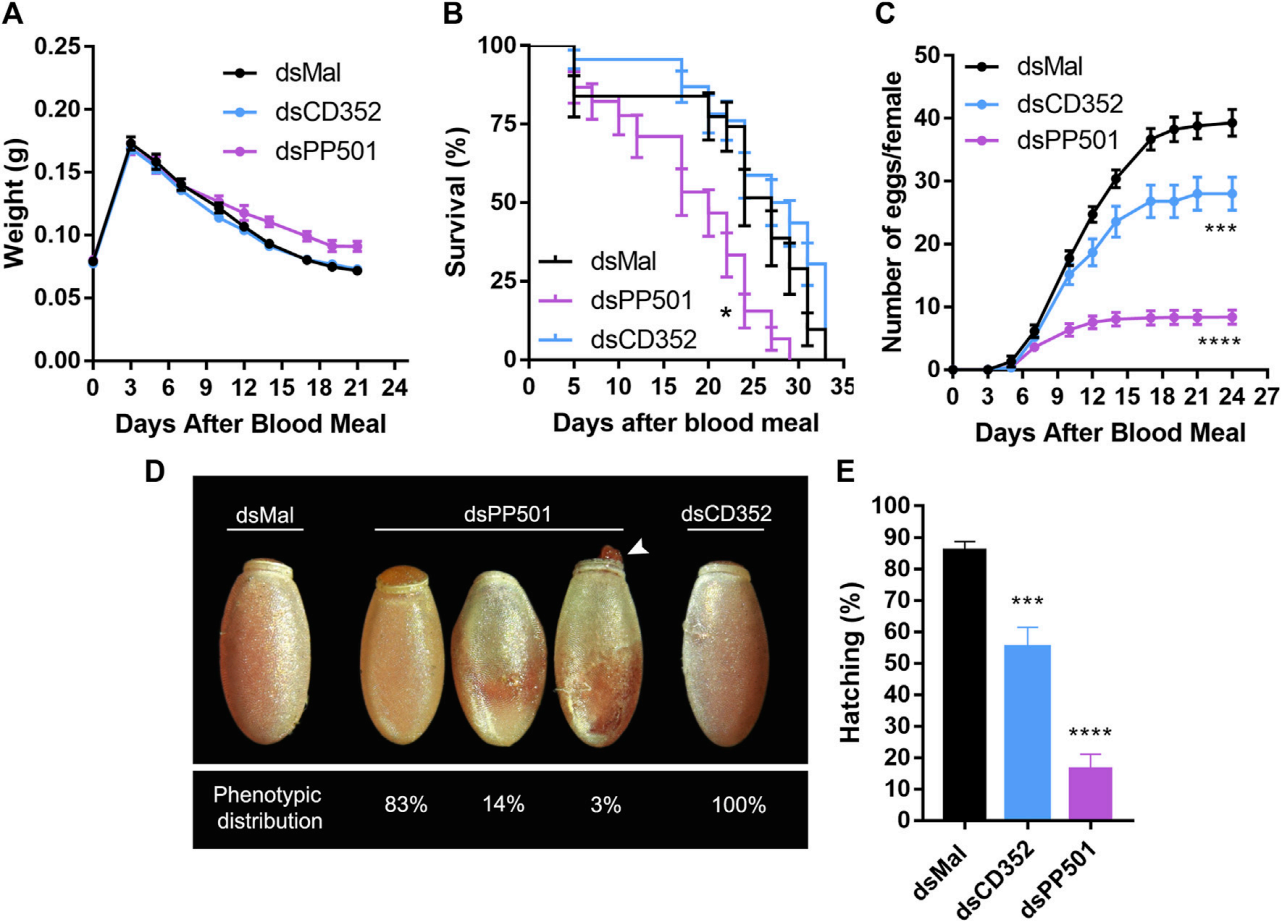
Silencing of PP501 and CD352 results in decreased oviposition and embryo lethality. **(A)**. Effect of PP501 and CD352 knockdown in the insect’s weight during digestion. Graph shows mean ± SEM (n = 45). Two-way ANOVA, *p* > 0.05. **(B)**. Survival curves of control and silenced females (n = 45). Graph shows mean ± SEM. *p < 0.05 Log-rank (Mantel-Cox) test. **(C)**. Number of eggs laid per female over 4 weeks after the blood meal. Graph shows mean ± SEM (n = 25). Two-Way ANOVA ***p < 0.001, ****p < 0.0001. **(D)**. Phenotypic distribution and representative images of the phenotypes observed in the F1 eggs laid by control and silenced females. **(E)**. Total hatching rates of F1 embryos from control and silenced females. Graph shows mean ± SEM (n = 25). ***p < 0.001, ****p < 0.0001. One Way ANOVA.

We chose to further investigate the functions of PP501 and CD352 during oogenesis due to their overall elevated expression patterns in the ovaries when compared to the other organs of vitellogenic females. Thus, double-stranded RNAs designed to target specific regions of the PP501 and CD352 mRNAs were injected into the insect’s hemocoel 2 days before the blood meal. Nine days later (7 days after the blood meal) the ovary, fat body, midgut, and flight muscle of control and silenced females were dissected, and their mRNA contents were analyzed by RT-qPCR. The PP501 expression levels were reduced by approximately 80% in all organs, with exception of the flight muscle, which was silenced by approximately 60% (Figure 2A). CD352 expression levels were decreased by approximately 95% in all organs (Figure 2B). Despite the systemic knockdown efficiency, no major alterations in the insect’s digestion were observed, as indirectly observed by measuring the insect’s weight after blood feeding (Figure 3A). Longevity, on the other hand, was slightly but significantly decreased in PP501-silenced insects when compared to controls (median survival of 27 days for control females and 20 days for silenced females, *p* < 0.05), and no changes were observed in CD352-silenced insects (median survival of 28 days) (Figure 3B). Interestingly, both PP501- and CD352-silenced insects presented decreased oviposition, with an average of 80% and 30% reduction when compared to controls, respectively (Figure 3C). In addition to the lower oviposition capacity, PP501-silenced insects laid an average of 17% of the eggs presenting some abnormal external morphology: 14% of them displayed uneven reddish color and a slightly altered external shape (Figure 3D), and 3% of them displayed a defective operculum structure presenting a perceptible leak of internal contents (Figure 3D, arrowhead), in addition to the uneven reddish color. In total, embryo viability was reduced by 30% in CD352-deficient F1 embryos and by more than 80% in PP501-deficient F1 embryos (Figure 3E). All eggs laid by PP501-silenced insects presenting the abnormal morphology phenotype (Figure 3D) were not viable (0% hatching rate), whereas the morphologically typical eggs laid by silenced females decreased their viability by 70% when compared to the levels observed in control eggs.

### Knockdown of PP501 triggers changes in the Ygs morphology

**FIGURE 4:**
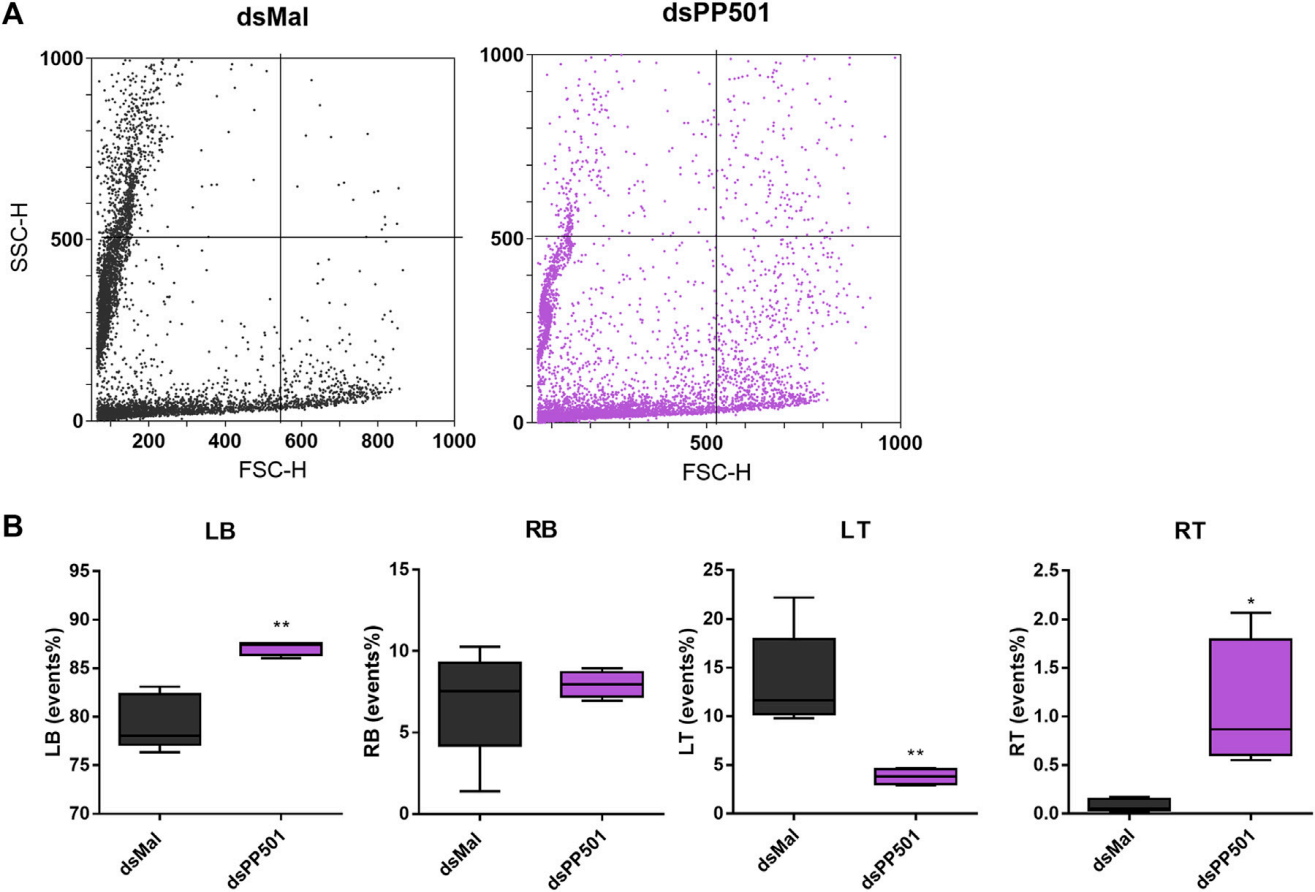
Knockdown of PP501 and CD352 results in abnormal distribution of the yolk organelles. **(A)**. Flow cytometry FSC x SSC dot-plots of the yolk organelles extracted from chorionated oocytes obtained from control and silenced females. The plots are representative of four experiments (n = 4). **(B)**. Quantification of the organelles percentage events (frequency) in each quadrant of the plots shown in **A** (RT, Right top; LT, left top; RB, right bottom; LB, left bottom). (n = 4) *p < 0.05, **p < 0.01, One Way ANOVA.

Because we found changes in the morphology of PP501-silenced eggs and high embryo lethality rates, we decided to further investigate the internal morphology of the PP501 unviable silenced eggs. To address this, we used flow cytometry to get further insight into the changes in the population of organelles in terms of size (FSC) and internal complexity (SCC), as previously performed (Vieira et al., 2021). Figure 4A shows representative dot-plots of the Ygs present in chorionated oocytes from control and silenced females. The representative plots allowed the identification of a broad range of events and showed that although the organelles are dispersed over a large range of sizes and internal complexities, the organelles from control and silenced samples presented different profiles as quantified in Figure 4B. For the silencing of PP501, there is an increase in the frequency of the Ygs in the right top quadrant (RT) as well as a decrease in the frequency of organelles in the left top quadrant (LT), indicating a shift to decreased complexity in smaller Ygs and increased complexity in a population of larger Ygs.

### Knockdown of PP501 impairs the activation of yolk degradation

**FIGURE 5:**
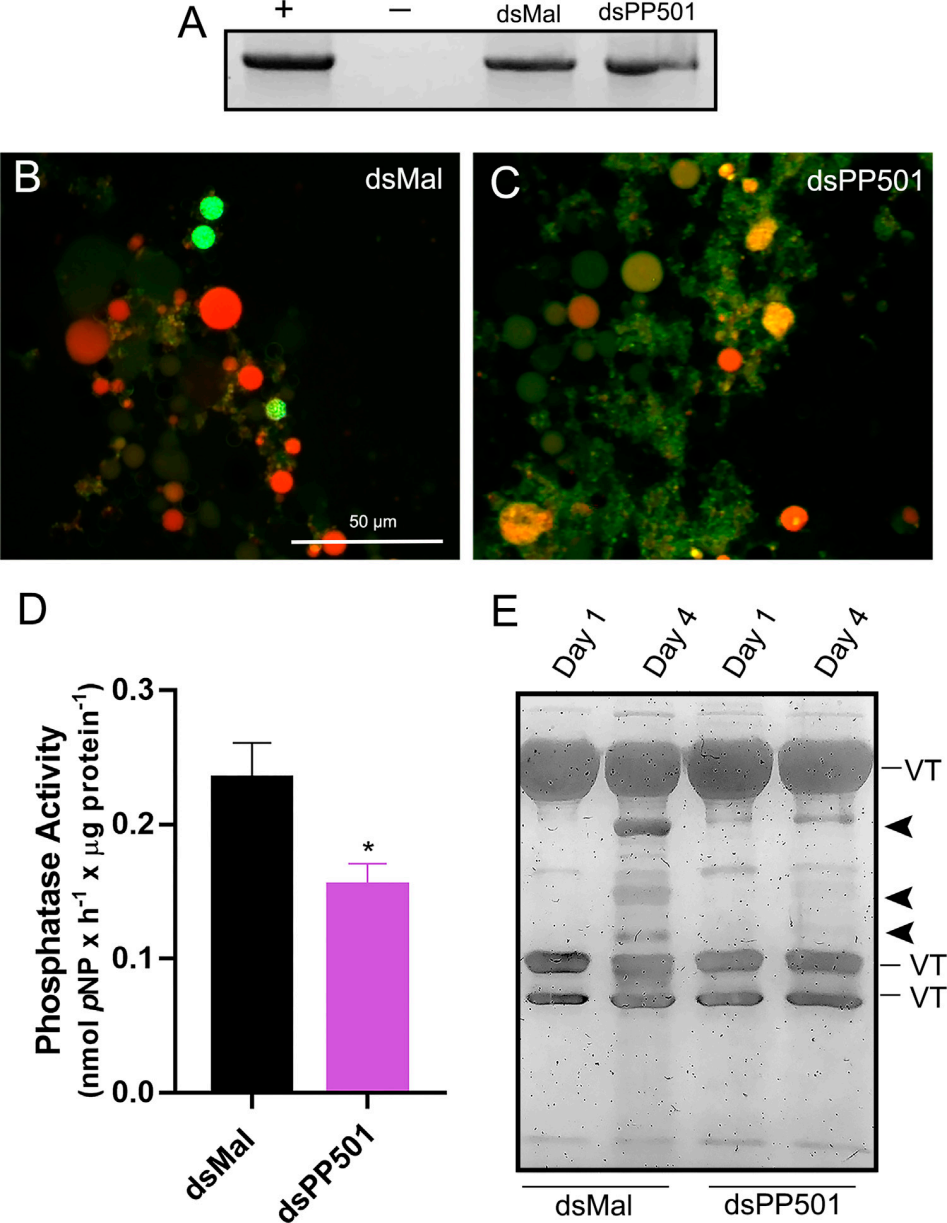
The silencing of PP501 does not alter fertilization but decreases Ygs acidification, acid phosphatase activity and impairs yolk mobilization. **(A)** genomic DNA from eggs laid by control (dsMal) and silenced (PP501) females were extracted and used as templates for amplification of a specific Y chromosome fragment by PCR. Genomic DNA from non-fertilized eggs laid by virgin females was used as negative controls (−). Fat body DNA from adult male insects was used as a positive control (+) (n = 2). **(B, C)**. Ygs suspensions extracted from day-4 eggs were incubated with the acidic environment marker acridine orange. The endogenous Ygs acidification was monitored in Ygs obtained from control (dsMal) and silenced (PP501) eggs. Images are representative of 3 experiments (n = 3). **(D)**. Specific activity levels of acid phosphatase in the egg homogenates at day-4 after oviposition. Graph shows mean ± SEM. (n = 8) *p < 0.05, *t*-test. **(E)**. 10% SDS-PAGE showing the protein profile of day-1 and day-4 eggs from control and silenced females. VT, Vitellin subunits. Arrowheads, products of VT degradation (n = 3).

To further investigate the outcomes of the previously observed abnormal Ygs biogenesis, and because none of the abnormal eggs laid by PP501-silenced insects presented visible signs of embryo development, we decided to investigate the main readouts known to be essential for the activation of the yolk degradation at early embryogenesis. In *R. prolixus*, it has been shown that fertilization and Ygs acidification are essential triggers to activate the yolk-targeting acid phosphatase activity during early embryogenesis (Nussenzveig et al., 1992; Fialho et al., 2002; Fialho et al., 2005). Thus, we tested the fertilization of the eggs using a marker of the Y chromosome as previously performed (Walter-Nuno et al., 2013), and found that PP501-silenced eggs were fertilized (Figure 5A). Eggs laid by virgin females were used as a negative control and the fat bodies of male insects were used as positive controls (Figure 5A). To further investigate the cause of impaired embryo development, we tested the Ygs acidification by incubating the organelles with the acid marker acridine orange (Ramos et al., 2007; Ramos et al., 2011). We found that silencing of PP501 partially impaired the acidification of the Ygs in day-4 silenced eggs when the onset of the endogenous acidification of the Ygs during embryogenesis occurs (Figures 5B, C). Furthermore, we found that the silencing of PP501 resulted in a 40% decrease in the activity of the yolk-targeting enzyme acid phosphatase (Figure 5D), and that, accordingly, the products of yolk protein degradation, which are visible starting on the fourth day of development (Figure 5E dsMal, arrowheads), are not generated in PP501-silenced eggs (Figure 5E).

## Discussion

In underdeveloped nations, the high frequency of neglected vector-borne illnesses like Chagas disease and dengue fever imposes significant health and financial burdens. The most important efficient management strategies for such illnesses have relied on vector population control throughout history and continue to do so now. Although early efforts in understanding the biology of specific vectors led to significant advances in the creation of management strategies for vector-borne diseases, studies regarding the intricate physiology of local vector species were hindered by the growing use of insecticide-based tools, which, for a while, were established to be both simpler and more efficient. The necessity to rely on in-depth species-specific vector biology has been highlighted once again by the growing threat of pesticide resistance and climate change, which can expand endemic areas (Eberhard et al., 2020; Wilson et al., 2020; Ramos and Gomes, 2022). Thus, disrupting molecular processes or attacking the metabolic targets required to generate viable eggs and/or embryos is one promising approach to managing vector populations.

Although the basic biology of yolk degradation is well understood and largely conserved, its molecular mechanisms have not yet been fully investigated. In *R. prolixus*, the recent completion of various genome and transcriptome sequencing projects (Medeiros et al., 2011; Ribeiro et al., 2014; Mesquita et al., 2015; Marchant et al., 2016; Leyria et al., 2020b; Leyria et al., 2020a; Coelho et al., 2021; Pascual and Rivera-Pomar, 2022) are likely to enable studies that not only expand our understanding of reproductive processes but also make it easier to identify new species-specific vector control targets. Here, we identified mRNAs encoding a protein phosphatase (PP501) and two aspartic proteases (CD352 and CD405) presenting high RPKM levels in the transcriptome of mature (chorionated) oocytes (Coelho et al., 2021), and performed functional tests to explore their role during oogenesis and embryo development. It is important to note that we decided not to investigate the functional role of CD405 in this study due to its higher expression in the midgut. Non-etheless, this does not rule out its potential a role in oocyte formation and embryo development, and further research into CD405s specific functional roles is likely worthwhile.

Using RT-qPCR, we observed that although PP501 and CD352 presented a tendency of higher expression levels in the ovaries, all mRNAs were also considerably detected in the other major organs of the adult insect (midgut, fat body, and flight muscle), indicating that the functional roles of those genes are not restricted to oocyte development. Within the ovary, all mRNAs tended to be more expressed in the tropharium, suggesting that this site is indeed the main source of maternal mRNA synthesis in the ovary and that cytoplasmic bridges are used to deliver the mRNAs to the transcriptionally inactive developing oocytes (Kunkel, 1991). This pattern, of highest expression levels in the tropharium, has been observed before in *R. prolixus* for genes of the autophagy pathway (Vieira et al., 2018; Bomfim and Ramos, 2020; Pereira et al., 2020), the ubiquitin-proteasome system (Pereira et al., 2022) and the unfolded protein response (Rios et al., 2022). It is important to notice, however, that although the temperature, humidity, light/dark cycles, and blood feeding intervals for each insect in this study were strictly controlled, it is expected that adult insects develop certain phenotypes during their early life. We observed significant variability in gene expression measured for each insect, even among control groups and regardless of the organ. On one hand, if one wants to extend the findings gained in the laboratory to wild animals in the field, it is vital to consider the fundamental variances among adult females of one species, which are reflected in this variable profile. On the other hand, such large variances make the statistical tests used to check for differences between conditions less reliable. Still, despite the high individual variance (and the absence of statistical significance in some of the data), both PP501 and CD352 presented a tendency of higher expression in the ovary, and, within the ovary, all transcript showed a tendency of higher expression in the tropharium.

Although parental RNAi triggered a systemic and efficient mRNA silencing of both PP501 and CD352, no major changes in blood protein digestion and lifespan were observed, whereas both knockdowns resulted in reduced oviposition and hatching rates, pointing to an essential requirement for oogenesis and embryogenesis to take place. In this regard, the PP501 phenotypes were especially robust, reducing the oviposition and embryo viability by approximately 80%. For this reason, we decided to further explore the phenotypes generated by the silencing of PP501.

Our findings that the chorionated oocytes produced by PP501-silenced females presented changes in the internal morphology of the Ygs population further demonstrate that, somehow, the silencing of this gene affected the regulations encompassing the biogenesis of the yolk organelles, and possibly the sorting of the yolk components. However, when comparing control and PP501-silenced day-1 eggs, the overall accumulation of the primary yolk protein VT was not changed (as seen in Figure 5E), demonstrating that, even though global oviposition was decreased, the eggs produced by PP501-silenced females were still able to accumulate adequate levels of yolk reserves. Interestingly, no signs of embryo development were observed in the eggs that failed to hatch, thus motivating us to investigate the possible reasons for the disruption of embryogenesis at such early stages. To address this, we tested if the silenced eggs were fertilized and, if yes, if the yolk degradation program was being properly activated. The checkpoints of the yolk degradation route are 1) fertilization, 2) Ygs acidification, 3) increase in acid phosphatase activity, and 4) yolk protein (mainly VT) degradation (Oliveira et al., 1989; Fialho et al., 2002; Ramos et al., 2007; Ramos et al., 2022). Interestingly, we found that, although the silenced eggs were fertilized, PP501-silenced eggs presented impaired Ygs acidification and lower acid phosphatase activity levels at day 4 of development, a time point when both events are known to be activated (Fialho et al., 2002; Fialho et al., 2005). According to the recognized essentiality of the acid phosphatase activity to trigger yolk degradation, the silencing of PP501 resulted in drastically impaired yolk protein degradation. Although causality is difficult to prove in this type of experiment, the possibility that the failure to trigger yolk degradation was at least one of the reasons why the early development was interrupted in PP501-silenced embryos cannot be ruled out. Altogether we found that PP501 is essential for the unfolding of the yolk degradation program and embryo development, and further studies on the specific functional roles of this (and other) genes will likely contribute to deepening our knowledge regarding this vector’s molecular reproductive biology.

## Data Availability

The original contributions presented in the study are included in the article/Supplementary Material, further inquiries can be directed to the corresponding author.
