# Supplementary material for: Functional characterization of maternally accumulated hydrolases in the mature oocytes of the vector Rhodnius prolixus reveals a new protein phosphatase essential for the activation of the yolk mobilization and embryo development
Source: Front Physiol. 2023 Feb 27;14:1142433. doi: 10.3389/fphys.2023.1142433 (PMC10008894; doi:10.3389/fphys.2023.1142433)
Supplement: Supplementary file 1 [file Table1.docx]

**Table S1: Genes and primers List.** All sequences were obtained from *Vector Base* (<https://www.vectorbase.org/>) and primers were synthesized by Macrogen or IDT technologies. T7 promoter sequence is underlined.

| **GENE** | **VECTOR BASE** | **PRIMER SEQUENCE (5’-3’)** | **AMPLICON**  **(bp)** | **PRIMER EFFICIENCY**  **(%)** |
| --- | --- | --- | --- | --- |
| **18S** | RPRC017412 | FOR: TCGGCCAACAAAAGTACACA  REV: TGTCGGTGTAACTGGCATGT | 105 | (Majerowicz et al., 2011) |
| **CD352 (qPCR)** | RPRC006759 | FOR: GCTTCACGTTCGTCAACTTATG  REV: CAGGCCTACTACGCCATCTACT | 195 | 83,187 |
| **CD352 (dsRNA)** |  | FOR: TAATACGACTCACTATAGGGTACTCCTGGTGTACTGTTTTACTC  REV: TAATACGACTCACTATAGGGTACTAGGGCATAACACAGATGGTA | 395 | - |
| **AP501 (qPCR)** | RPRC002352 | FOR: GCACATCAGGTAGTGGAAGATG  REV: GGAAAGAACACATCAGGGTTTC | 142 | 96,099 |
| **AP501 (dsRNA)** |  | FOR: TAATACGACTCACTATAGGGTACTGAGCGAAACAGAAGTAAGAG  REV: TAATACGACTCACTATAGGGTACTCATCCGTAGGTCTCATAATC | 493 | - |
| **CD405 (qPCR)** | RPRC006028 | FOR: CAGGAAGCGATAATCTCGAAG  REV: GGGAATGGACCATCTATACCAA | 138 | 121,604 |
| **Y chromosome genomic sequence** | ACPB03041887  GenBank: JX559072.1 | FOR: TCCTCCGCCTTGCTTCTCTGT  REV: GTGCGGGCGGTGGATTG | 894 | - |

Majerowicz, D., Alves-Bezerra, M., Logullo, R., Fonseca-De-Souza, A. L., Meyer-Fernandes, J. R., Braz, G. R. C., et al. (2011). Looking for reference genes for real-time quantitative PCR experiments in Rhodnius prolixus (Hemiptera: Reduviidae). *Insect Mol. Biol.* 20, 713–722. doi:10.1111/j.1365-2583.2011.01101.x.
