# Supplementary figures and images for: Functional characterization of maternally accumulated hydrolases in the mature oocytes of the vector Rhodnius prolixus reveals a new protein phosphatase essential for the activation of the yolk mobilization and embryo development
Source: Front Physiol. 2023 Feb 27;14:1142433. doi: 10.3389/fphys.2023.1142433 (PMC10008894; doi:10.3389/fphys.2023.1142433)

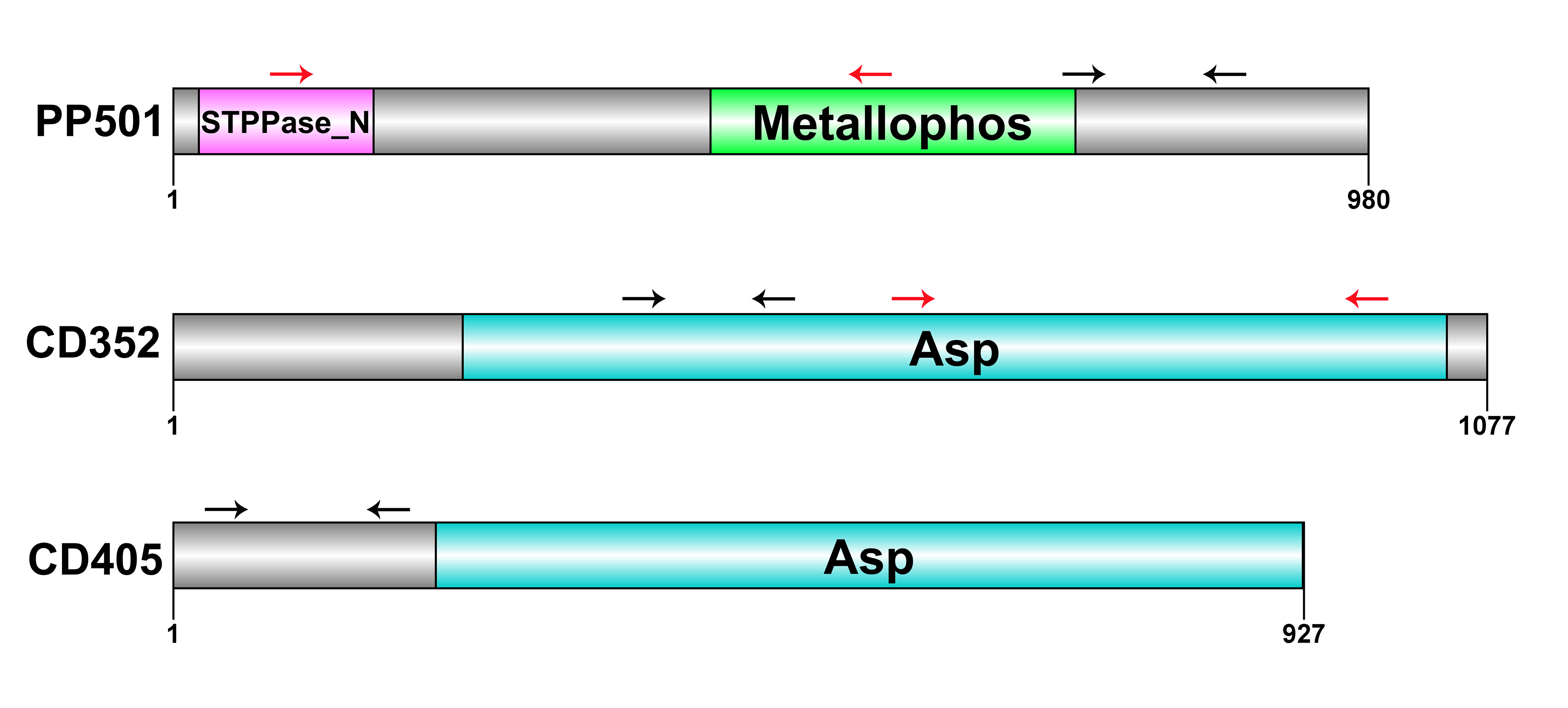

Supplement: Supplementary file 2 [file Image2.TIF]

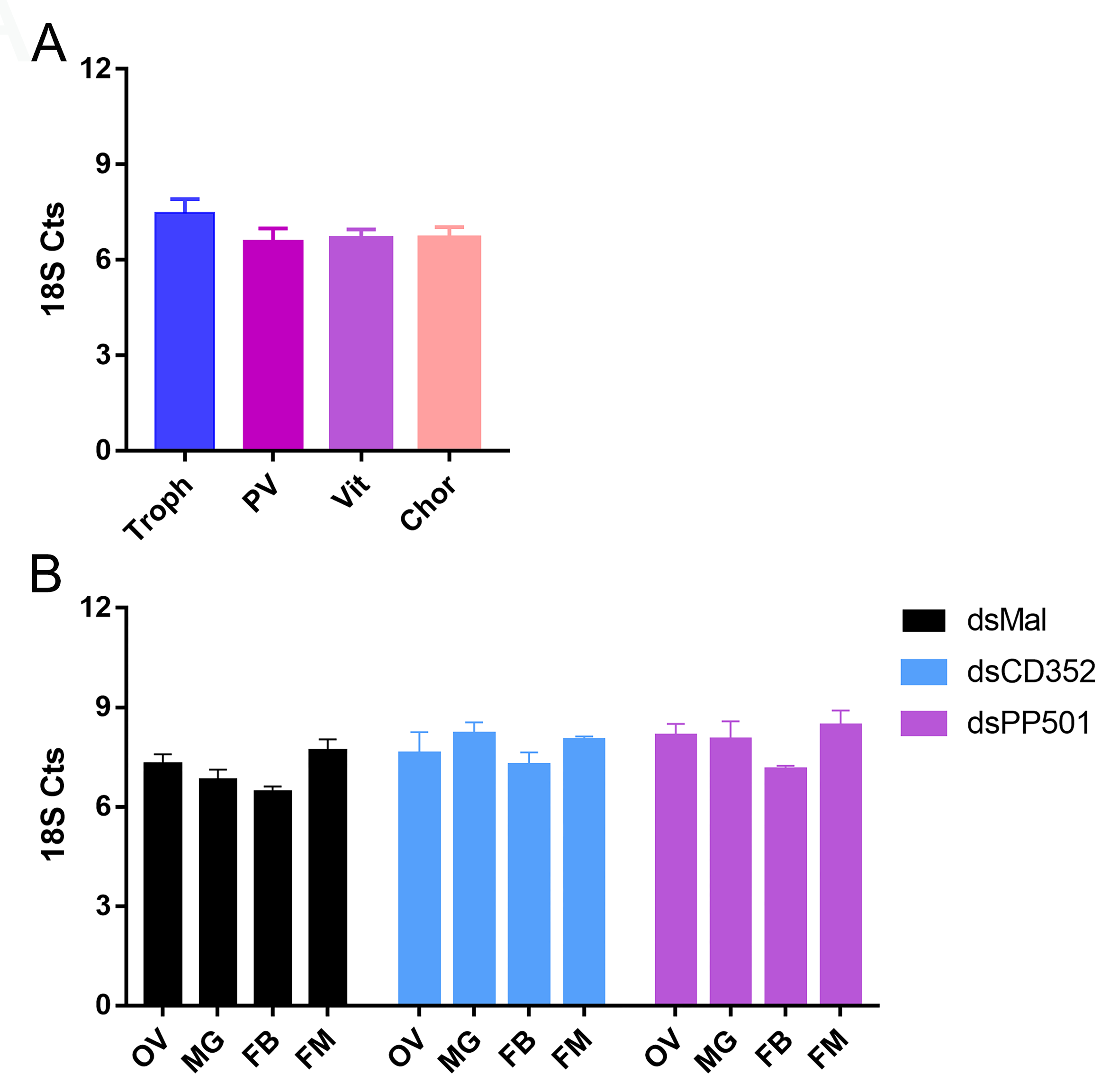

Supplement: Supplementary file 3 [file Image1.TIF]
